# On the Impact of Localization and Density Control Algorithms in Target Tracking Applications for Wireless Sensor Networks

**DOI:** 10.3390/s120606930

**Published:** 2012-05-25

**Authors:** Andre N. Campos, Efren L. Souza, Fabiola G. Nakamura, Eduardo F. Nakamura, Joel J. P. C. Rodrigues

**Affiliations:** 1 Instituto de Computação, Universidade Federal do Amazonas, Av. Rodrigo Otavio, 6200, Campus, Setor Norte, CEP 69077-000, Manaus, AM, Brazil; E-Mails: andre.campos@acm.org (A.N.C.); efren@icomp.ufam.edu.br (E.L.S.); fabiola@icomp.ufam.edu.br (F.G.N.); 2 Analysis, Research and Technological Innovation Center (FUCAPI), Av. Danilo Areosa, 381, Distrito Industrial, 69040-420, Manaus, AM, Brazil; 3 Instituto de Telecomunicações, Universidade da Beira Interior, Av. Marquês D'ávila e Bolama, 6201-001, Covilhã, Portugal; E-Mail: joeljr@ieee.org

**Keywords:** target tracking, integrated algorithms, density control, localization

## Abstract

Target tracking is an important application of wireless sensor networks. The networks' ability to locate and track an object is directed linked to the nodes' ability to locate themselves. Consequently, localization systems are essential for target tracking applications. In addition, sensor networks are often deployed in remote or hostile environments. Therefore, density control algorithms are used to increase network lifetime while maintaining its sensing capabilities. In this work, we analyze the impact of localization algorithms (RPE and DPE) and density control algorithms (GAF, A3 and OGDC) on target tracking applications. We adapt the density control algorithms to address the *k*-coverage problem. In addition, we analyze the impact of network density, residual integration with density control, and *k*-coverage on both target tracking accuracy and network lifetime. Our results show that DPE is a better choice for target tracking applications than RPE. Moreover, among the evaluated density control algorithms, OGDC is the best option among the three. Although the choice of the density control algorithm has little impact on the tracking precision, OGDC outperforms GAF and A3 in terms of tracking time.

## 1. Introduction

A Wireless Sensor Network (WSN) consists of a set of sensors distributed over an area of interest and capable of collecting information from the environment. Therefore, the correct localization of these events and the sensors themselves is necessary for a proper analysis of the gathered data [1]. The position of the sensors cannot be determined beforehand. Only a fraction of these sensors can determine their location; these nodes are called *beacons*. The remainder nodes are known as *free nodes*. The beacon nodes assist free nodes to estimate their unknown positions.

The sensors also have limited service life; they use batteries as the only energy supply and recharging them is often impossible [2]. Therefore, battery usage directly impacts the network lifetime. Density control algorithms can be used to save energy by keeping only a fraction of the nodes enabled while leaving the rest in stand-by mode and maintaining sensing coverage of the area of interest.

These two problems, namely localization and density control, play fundamental roles in target tracking applications, which is an important application of WSNs. For example, the SAUIM project aims at tracking an endangered species of monkeys in the jungle. The continuous and long-term tracking can help us to protect these animals, since it allows a thorough study of their habits. The success of that project depends on the network's ability to operate for as long as possible and on the tracking accuracy.

Although these problems are important, they are usually solved separately. However, deploying a sensor network demands to integrate different solutions for different layers, such as application, routing, medium access, density control, synchronization, and localization. In this integrated approach, Souza *et al.* [3] show how localization algorithms impact on target tracking; Oliveira *et al.* [4] offer a similar evaluation considering geographical routing and localization algorithms; and Siqueira *et al.* [5] integrate routing and density control algorithms and show the benefit of such an integration.

A major contribution of this work is the adaptation of the density control algorithms (GAF, OGDC and A3) for the more general *k*-coverage problem (every point in the sensor field is covered by at least *k* sensors). A second contribution is the use of a localization-error estimation metric to select which nodes should be turned off. Given these contributions, we assess the target tracking performance of a wireless sensor network when density control is applied and localization errors exist. As a proof-of-concept, we evaluate how different combinations of localization algorithms (RPE and DPE) and density control algorithms (OGDC, GAF and A3) impact target tracking (the modified algorithms are used). To the best of our knowledge, there is no work in the literature that combines localization, density control and target tracking into the same scenario.

The remainder of this paper is organized as follows. In Section 2, we present the related work. Section 3 presents the theory behind the localization and density control algorithms. Section 4 discusses modifications on the density control algorithms that we propose. A detailed discussion of our methodology and results is presented in Section 5. Finally, Section 6 brings our lessons learned and recommendations.

## 2. Related Work

The localization problem has been widely discussed in WSN. Niculescu and Nath [6] propose the Ad Hoc Positioning System (APS). In APS, the free nodes use the number of hops to the beacons to estimate the distance between them; this technique is known as DV-Hop. The algorithm works with a low number of beacons and low density. However, its applicability is questionable due to its high localization errors and communications costs [7]. Boukerche *et al.* [7] propose an algorithm called DV-Loc. This algorithm also uses DV-Hop to estimate distances between beacons and free nodes. The difference is that the scope of the flooding is constrained to cells of a Voronoi Diagram. As a result, the algorithm is more scalable and precise than APS. Albowicz and Zhang [8] present a recursive solution for the problem called Recursive Position Estimation (RPE). This is a common baseline for localization algorithms, and it is further discussed in Section 3.1. Oliveira *et al.* [9] present a variation of RPE, called Directed Position Estimation. A major difference is that DPE needs only four beacon nodes organized in a predefined structure. Due to its consistent improvements, we also evaluate the DPE algorithm, which is described in Section 3.1.

As far as the density control problem goes, Ye *et al.* [10] propose Probing Environment and Adaptive Sleeping (PEAS). This algorithm consists in waking up each node periodically to determine if its neighbor must be replaced; its limitation is the premise that the “node density must be much higher or even orders of magnitude higher than the minimum number of nodes needed for normal operation”. Cerpa and Estrin [11] propose Adaptive Self-Configuring sEnsor Network Topologies (ASCENT), where each node decides whether or not it participates on the topology according its connectivity level with the network. Xu *et al.* [12] propose the Geography-informed energy conservation for Ad Hoc routing (GAF) algorithm, a pioneer solution for density control. Together with the Optimal Geographical Density Control (OGDC) [13], GAF is a key baseline for comparing density control algorithms based on localization data. The A3 [14] does not depend on localization data, which represents another class of density control algorithms. Due to their importance, GAF, OGDC and A3 are evaluated (and described in Section 3.2).

Target tracking is one of the most importation applications of WSN. Schmitt *et al.* [15] use *maximum a posteriori* (MAP) to allow a team of robots to estimate their collective localization in a known environment and track moving objects. Yang *et al.* [16] proposes the use of moving averages to reduce the tracking error. Doǧançay and Hashemi-Sakhtsari [17] apply a technique based on the Least-Squares method to reduce the tracking error. According to Sorenson [18], this technique evolved and eventually turned into the Kalman Filter (KF) [19]. Gordon *et al.* [20] brought a new approach when he introduced the Particle Filter (PF). Currently, the KF and PF are the most popular techniques for target tracking in WSN.

The original Kalman Filter works well with linear models when the error can be modeled as Gaussian noise [21]. To overcome such a limitation, extensions like the Extended Kalman Filter (EKF) [22] and the Unscented Kalman Filter (UKF) [23] have been introduced. According to Nordlund and Gustafsson [24], if the system is not linear or the noise is not Gaussian, the Particle Filter is more appropriate. For instance, Aslam *et al.* [25] propose a solution that combines binary proximity sensors and a particle filter for target tracking.

Taking a broader approach, Gui and Mohaptra [26] analyze the impact of different density control algorithms in target tracking applications; the work focuses on keeping the original coverage while maximizing the network lifetime. Souza *et al.* [3] compare the tracking errors from KF and PF when the localization errors resulting from RPE and DPE are taken into consideration. Oliveira *et al.* [4] investigate the effects of localization errors on geographical algorithms like routing and density control. However, we still need to investigate the impact of density control on routing algorithms that do not depend on localization [27–30].

In this work, to provide an integrated evaluation, we adapt density control algorithms for the more general *k*-coverage problem. We also propose the use of a localization-error estimation metric to select the active nodes. Then, we show how target tracking algorithms are affected by density control and localization errors. As a proof-of-concept, we assess different combinations of localization algorithms (RPE and DPE) and density control algorithms (OGDC, GAF and, A3). The localization algorithms and the density control algorithms were carefully chosen. Those are reference baselines for most of the current solutions, hence, they allow us to define a common reference for the sake of comparison. In addition, the proposed modifications should be easily extended to other algorithms.

## 3. Background

### 3.1. Localization

In some applications, the nodes of a WSN must estimate their own positions. However, it is not always possible to equip all sensors with a GPS. Therefore, only a fraction of the nodes are equipped with such capability; these nodes are called beacons. Through the combination of the positions from the beacons and localization algorithms, the other nodes (called free nodes) are capable of estimating their positions. This process can be divided into three steps [1]: distance estimation, position computation, and localization.

The distance estimation can be done through Received Signal Strength Indicator (RSSI) or some variation of Time of Arrival (ToA) [1]. RSSI consists in estimating the distance based on the power of the received signal. Although it is sensitive to interference and physical obstacles between the nodes, this method is cheap in terms of hardware and energy consumption. ToA consists in measuring the time a message takes to travel from node *A* to node *B*, which requires synchronization between the sensors and thus increases costs.

After estimating the distance to a minimum set of beacons, a free node is capable of calculating its position. Methods like trilateration or multilateration can be used. Trilateration is essentially a geometry problem: estimate the position of the free node based on the coordinates of three references and the distances from these references to the free node. Although it is intuitive, trilateration does not work in practice due to the imprecision on the distance estimations. Multilateration, on the other hand, uses a larger number of reference nodes to create an overdetermined system of equations; this system is solved with optimization methods, such as Least Squares.

The last stage of the localization system is to use a localization algorithm; this work evaluates two of these algorithms: the Recursive Position Estimation (RPE) [8] and the Directed Position Estimation (DPE) [9].

#### 3.1.1. Recursive Position Estimation

**Figure 1.**
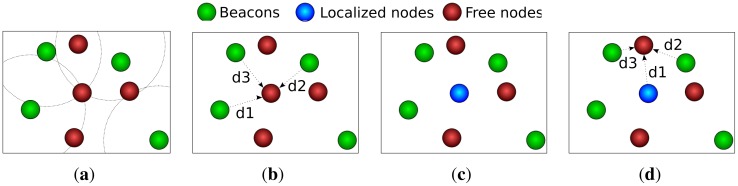
Stages of *Recursive Position Estimation*. (**a**) Beacon Selection; (**b**) Distance Estimation; (**c**) Position Estimation; (**d**) New Beacon.

RPE is divided into four stages, as depicted in Figure 1. In stage one, the free node establishes its reference nodes. After that, the node estimates its distance to the beacons using RSSI or ToA. Subsequently, the node estimates its own position through multilateration. Finally, if the node has enough confidence on its position estimation, it behaves like a beacon and helps other nodes on the localization process. The localization confidence is estimated through a parameter called residual, as follows:

(1) $$\text{residual}\left(\text{x},\text{y}\right)=\sum_{i\in \text{references}}{\left(\sqrt{{\left(x_{i}-x\right)}^{2}+{\left(y_{i},y\right)}^{2}}-d_{i}\right)}^{2}$$

where (*x_i_, y_i_*) is the position of the *i*th reference node and *d_i_* is the average distance measured (using RSSI or ToA, for example).

RPE makes the number of reference nodes grow rapidly; this is its main strength. However, there is a side effect: the localization errors are also propagated quickly [4]. Experiments show that RPE needs at least 5% of the nodes to be beacons; the algorithm is capable of localizing 90% of the nodes with an error up to 3% of the distance between the nodes [8].

#### 3.1.2. Directed Position Estimation

**Figure 2.**
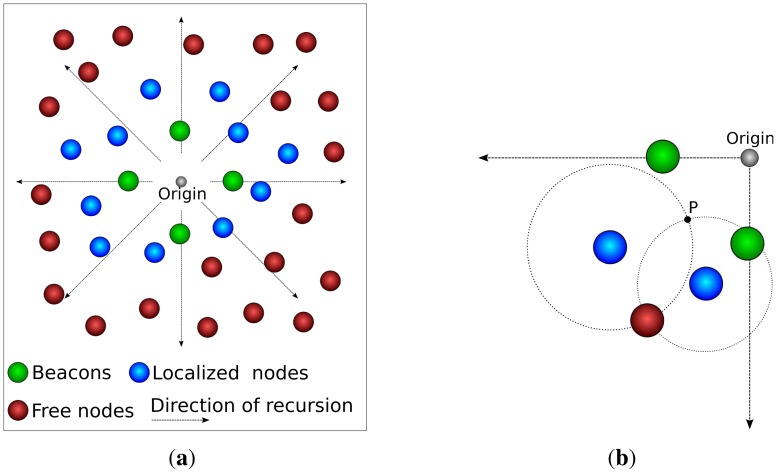
Stages of the Directed Position Estimation. (**a**) DPE Overview; (**b**) Trilateration with DPE.

DPE is a variation of RPE. The main difference is that DPE uses only 4 beacons, arranged in a cross-like structure. The use of such structure guarantees that the recursion has only one origin, as depicted in Figure 2(a); that means the direction of the propagation of the recursion is known (it goes from the center of the cross-like structure towards the edges of the sensing area).

For the sake of our argument, let us consider that the distance estimation is perfect, which would allow us to use trilateration. To use trilateration, a node must know its distance to three reference nodes. If a circle is drawn from the node to each beacon (where the radius is the distance between them), the three circles will intersect at the node' location. Now let us consider the case where we use only two reference nodes. In this case we would have two intersection points for the circles, where one is the right position of the node and the other is not. Since we know the direction of the recursion (a DPE feature), the correct intersection is the farther from the recursion origin, as depicted in Figure 2(b).

### 3.2. Density Control

Density control consists in turning off as much sensors as possible for as much time as possible while keeping the network's functionality. This work evaluates *Optimal Geographical Density Control* (OGDC) [13], *Geography-informed Energy Conservation for Ad Hoc Routing* (GAF) [12], and A3 [14].

#### 3.2.1. Optimal Geographical Density Control

The idea behind this algorithm is that minimizing the intersection between the sensing areas of all sensors in the network implies optimal use of energy. This algorithm assumes that each sensor knows its position. In a nutshell, the algorithm is based on the mathematical proof that the minimal coverage disk intersection of three sensors is achieved when they are arranged as an equilateral triangle of sides √3*r*, where *r* is the sensing radius. It is impractical to have a network so dense that a sensor can always be found at an optimal position. Therefore, the sensors closer to the optimal points are used.

**Figure 3.**
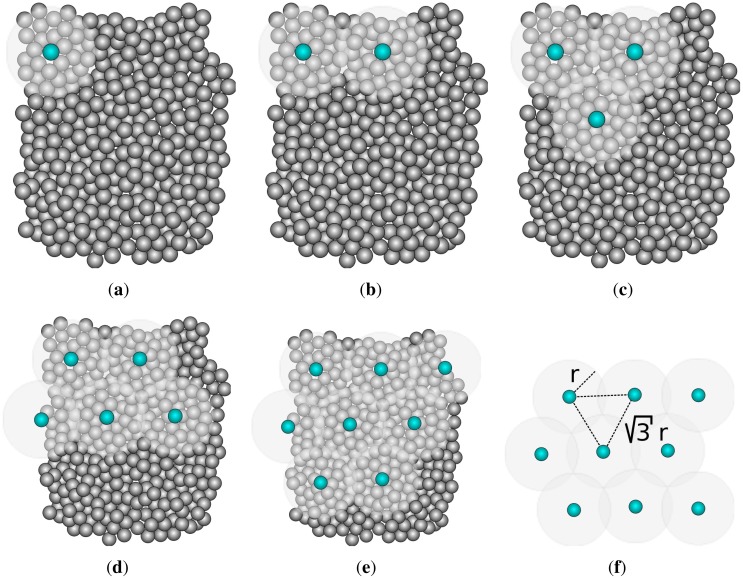
Overview of *OGDC*. (**a**) Algorithm starts; (**b**) Neighbor in random direction; (**c**) Equilateral triangle; (**d**) Process repeats; (**e**) Nodes cover sensing area; (**f**) Algorithm completed.

A topology is periodically created. Any node can start the algorithm (the choice is based on the remaining energy level). The algorithm starts with a node *A* selecting a random direction and broadcasting a message. A node *B* located on that direction and at a distance √3*r* from *A* joins the network (as shown in Figure 3). To cover one of the intersection points between the sensing disks of *A* and *B*, a third node *C* closer to the point where *A, B* and *C* form an equilateral triangle joins the network. This process repeats until all nodes are either enabled or standing by. The sensors remain enabled for a predetermined time. After that, the whole process restarts and other nodes have the opportunity to participate on the network.

Zhang and Hou [13] show that OGDC can use less than half of the nodes needed by a GAF variation while keeping the same sensing coverage.

#### 3.2.2. Geography-Informed Energy Conservation for Ad Hoc Routing

**Figure 4.**
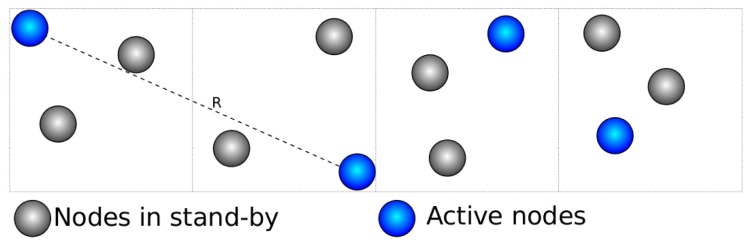
Geography-informed Energy Conservation for Ad Hoc Routing.

GAF consists in creating a virtual grid on the sensing area. Once a free node starts to know its position, it can determine its cell on the virtual grid. Nodes in the same cell are considered equivalent. The cell size is dimensioned such that any nodes in two adjacent cells can communicate. Therefore, the radio range must be greater than or equal to *R* in Figure 4. In other words, the cell size must be smaller than $\frac{R_{c}}{\sqrt{5}}$.

Nodes can operate in three modes: active, discovery and sleeping. All nodes start in discovery mode, which means that they are looking for equivalent nodes. Once the equivalent nodes have been identified, they follow an application-dependent protocol to decide which nodes will sleep and which node will stay active. After some time, sleeping nodes return to the discovery mode and the active node gives the opportunity to another node.

Xu *et al.* [12] show that GAF reduces the energy consumption by 40% to 60, compared to a network without density control. In addition, they show that the network lifetime increases with node density.

#### 3.2.3. A3

A3 uses an approximated solution of the Minimal Connected Dominating Set (CDS) problem, which is NP-Hard. This algorithm does not require the nodes to know their positions; it uses only the RSSI-based distance estimations to create a sub-optimal CDS tree. Any node can start A3 through a Hello Message; that makes the neighbors see it as a parent node through a Parent Recognition Message. Then, the parent node creates a list of its children and sorts the list by distance in descending order. This list is transmitted to the children; each child waits for a time proportional to its position on the list. If a child receives a Sleeping Message from another node in the list, it enters stand-by mode. In other words, if a node *A* can communicate with node *B* and both communicate with a parent *P*, the one closer to *P* will enter sleeping mode. After that, nodes that did not receive a sleeping message start the process as parents.

Wightman and Labrador [14] show that A3 only needs 6% of the nodes to be active when the network density is high (e.g., >0.2 sensors/m^2^). In low densities (e.g., <0.05 sensors/m^2^), it needs around 41% of active nodes. In addition, the number of messages sent is shown to increase linearly with network density.

## 4. Adaptation on the Density Control Algorithms

### 4.1. Sensing Coverage

Among the density control algorithms that we evaluated, only OGDC aims to keep the sensing coverage of the network; both GAF and A3 attempt to build a topology that ensures communication coverage. Given that we are interested in target tracking applications, we suggest a few modifications on GAF and A3 such that they build a network capable of sensing the entire area of interest. For both algorithms the adjustments are merely computational and do not result in higher costs.

#### 4.1.1. GAF

**Figure 6.**
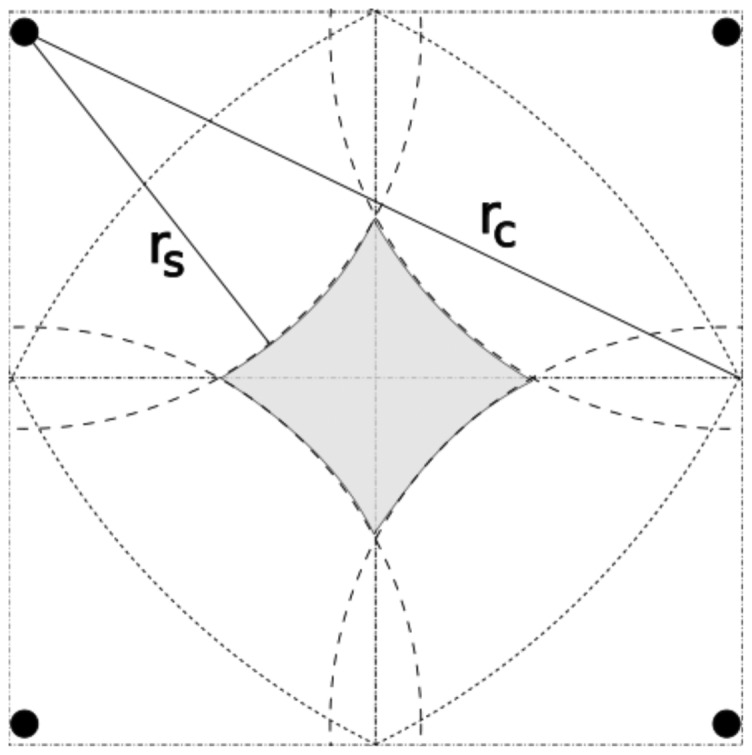
Sensing coverage with the original version of GAF.

If we use a cell size of $\frac{r_{C}}{\sqrt{5}}$, as suggested by the GAF authors, we could have a hole in sensing coverage, as shown in Figure 6. To avoid that, we can make the cells as wide as $\frac{r_{s}}{\sqrt{5}}$. Consequently, we guarantee that all neighbors detect the same target. For instance, a target located at cell (*i, j*) will be detected by sensors located at cells (*i, j*), (*i*−1, *j*), (*i*+1, *j*), (*i, j* −1), and (*i, j* +1). Intuitively, we could argue that this implies 5-coverage. In fact, the neighbors located at cells (*i* − 1, *j* − 1), (*i* − 1, *j* + 1), (*i* + 1, *j* − 1), and (*i*+1, *j* +1) detect the target, depending on the targets position. In this case we would actually have 7-coverage, as discussed in Section 4.2.

#### 4.1.2. A3

In the original algorithm [14], the sensor that initiates the algorithm sorts its neighbors in descending order by distance. Our modification consists in changing the sorting criteria; we use the distance to the sensing perimeter. That way, sensors located *r_s_* away from their parents are ranked higher than the ones located *r_s_* ± *ε* away.

### 4.2. Adding k as a Parameter

It is necessary to modify the density control algorithms such that it is possible to specify the minimum number of sensors that must cover all points of the area of interest (*k*-coverage). Our work assumes that 3-coverage is required for target tracking. In addition, we evaluate scenarios with 6-coverage and 9-coverage.

#### 4.2.1. OGDC

The modifications that needed to have *k* as a parameter in OGDC are discussed in the literature [13]. The modification consists of changing how a node verifies if its neighbors completely cover its sensing area. The mechanism utilized for that is not illustrated by the authors [13]. Intuitively, we could create a virtual grid centered on the sensor of interest. In the original algorithm, if all points of the grid are located at a distance less than or equal to *r_s_* from the center are also located at a distance less or equal to *rs* from any other sensor, we consider that the sensing disk of that node is covered by its neighbors (and that sensors can be turned off). In other words, we have 1-coverage.

For *k*-coverage, we need to guarantee (if possible) that every point in the sensor field is covered by at least *k* sensors. Thus, a node can only be turned off if its coverage area is overlapped by *k* sensors already. This modification on the algorithm has no computational or communication penalty in comparison to the original algorithm.

#### 4.2.2. GAF

To tune GAF to accept *k* as a parameter, it is necessary to modify the cell size. To determine an equation for *k* in function of the ratio between the cell size and sensing radius, we performed a series of simulations. After assembling 10,000 networks mimicking GAF with different cell sizes, we obtained the model:

(2) $$k=f\left(x\right)=41.486\times e^{-4.4713x}+1.3738$$

in which *x* is the ratio between the cell size and the sensing radius.

**Figure 7.**
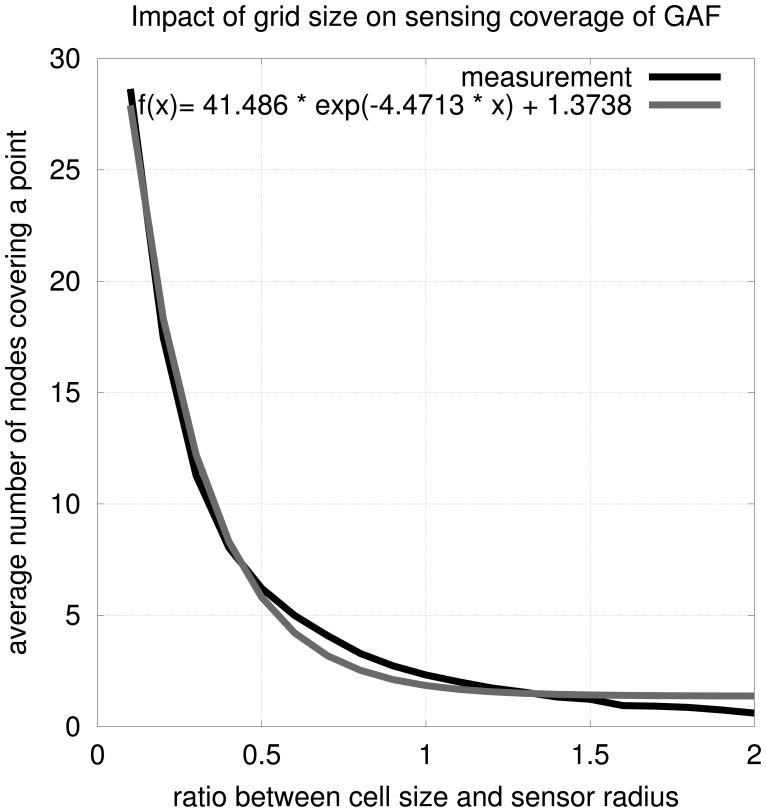
Determining GAF cell size with respect to *k*.

Rewriting the equation in function of *k* we have

(3) $$g\left(k\right)=\frac{1}{4.4713}\ln\left(\frac{k-1.3738}{41.486}\right)$$

in which *g(k)* is the ratio between cell size and sensing radius. This function is an exponential approximation of the data we gathered (see Figure 7); we used the Least Squares Method for that approximation. For instance, if we consider the scenario discussed in Section 4, where the cell size is $r_{s}/\sqrt{5}=0.447r_{s}$, by setting *x* = 0.447 in Equation (2), we obtain *k* ≈ 7. For *k* = 3, *g*(3) = 0.7244; *i.e.*, for 3-coverage, the cell size should be 0.7244*r_s_*. This modification is purely numerical and does not represent any difference in terms of computational and communication costs.

#### 4.2.3. A3

In the original algorithm, the time a node sleeps after receiving a child recognition message is proportional to its position on the list, which is sorted in descending order by distance. Our first modification discussed in Section 4 changes this criteria to the distance to the sensing perimeter. To make *k* a parameter for the algorithm, we need to make another modification to A3. Now, the time a node sleeps is given by the integer division of its position on the list by *k*. In other words, the *k* nodes closer to the sensing perimeter will become parent nodes. This modification does not imply higher computational costs. However, the communication costs go from *O*(*dv*) to *O*(*dkv*), where *d* is the number of density control rounds, *v* is the average number of neighbors and *k* is the number of sensors that must be able to detect the target (related to *k*-coverage).

### 4.3. Density Control with Residual

#### 4.3.1. OGDC

In OGDC, the sleeping time is proportional to the distance between the sensor's position and the ideal position. The mechanism we use for adding the residual is adding a period of time proportional to the residual to the time the sensor waits. Consequently, sensors with lower residual behave like in the original version of the algorithm. However, a sensor with high residual (*i.e.*, greater than 10) waits longer than in the original version, even when its estimated position is close to the ideal position. This modification has no impact on the communication and processing costs of the algorithm.

#### 4.3.2. GAF

The time a sensor must sleep now depends on the inverse of the residual. Thus, a sensor with a smaller residual value sleeps for less time and has a higher probability to participate in the network. In addition, the tie-breaker criteria for sensors in the same cell becomes the residual (instead of the node ID in the original version). Again, these modifications have no impact on the communication and processing costs of the algorithm.

#### 4.3.3. A3

The A3 Algorithm assembles a topology where the enabled nodes are the ones farther away from the parents. The modification presented in Section 4 suggests that the distance from the children to the parents' sensing perimeters can be used as the sorting criterion. To use the residual error in this process, we suggest a radical conceptual change (but computationally simple). The idea is to create a topology that minimizes the residual; the sorting criterion is the residual of each node. In other words, each sensor that starts the process essentially selects its *k* neighbors with smaller *residuals* to participate in the network (where *k* is also a parameter, as discussed in Section 4). This set of modifications incur no penalty on the computational or communication costs.

## 5. Performance Evaluation

### 5.1. Methodology

**Figure 8.**
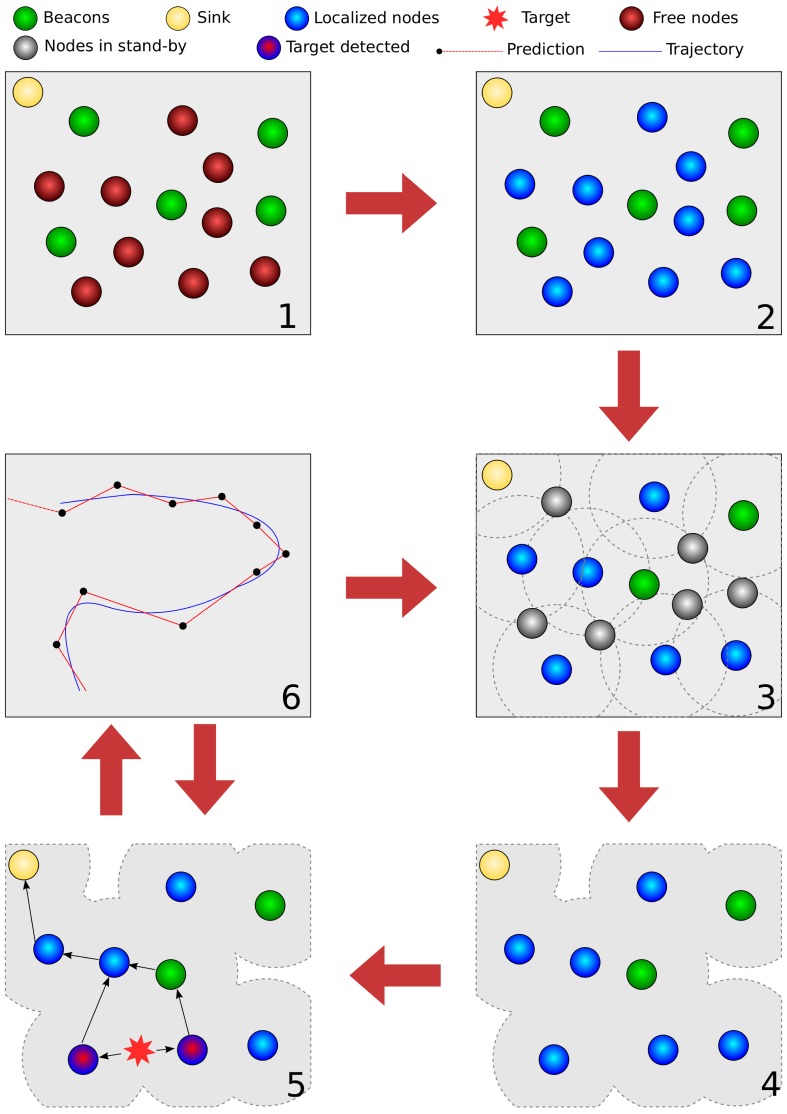
Methodology.

Figure 8 depicts our experiments. At first, sensors are uniformly randomly distributed over the area of interest. After that, a localization algorithm is applied, followed by a density control algorithm (steps 3 and 4). Subsequently, the enabled nodes start to sense the target and send tracking messages to the sink node (steps 5 and 6). The process periodically goes back to step 3 so that other sensors can participate in the network; this period is application-dependent.

The experiments are performed in the Sinalgo simulator [31], which is a Java-based network simulator. Tools like NS-2 simulate the transport, MAC and physical layers of the network. Sinalgo, on the other hand, focuses on validating network algorithms; it abstracts the complexity of the deeper layers of the network. Its learning curve and its platform independence were the decisive factors for its adoption.

Time is discrete in Sinalgo and is based on rounds. We use the approximation that a round is equivalent to 10 ms. The duration of one simulation can go up to 100,000 rounds (or 1000 s). The simulation terminates if that limit is reached or if the 3-coverage remains below 60% for a time larger than a density control round. A density control round is the time it takes for a density control algorithm to execute again. We set this time to be 7500 rounds (or 75 s) in our experiments.

The imprecision on the distance estimation between two sensors *A* and *B* is simulated with a Gaussian noise of mean 0 and variance 0.01*d*, where *d* is the Euclidean distance between *A* and *B*. The energy consumption model we used is similar to the one used by Xu *et al.* [12]: if the energy needed to keep a sensor active for one round is 1.0 J, then the node needs 1.6 J to send one message and 0.0025 J to be in stand-by mode. All nodes receive enough energy to remain active without sending any messages for 25,000 rounds.

Random walk [32] is often used as a mobility model in simulations. However, it is a stateless model; the motion shows sudden changes in both direction and acceleration. Therefore, we chose to use a mobility model called Correlated Random Walk (CRW) [33]. In this model there is a correlation between successive steps of the target's motion. Our implementation of the model uses a correlation parameter of 0.999 and distance scaling parameter of 0.08. As a result, the target simulates an animal moving at 3.9 m/s.

In the results discussion, we consider the following metrics:

- *k*-**coverage** measures the percentage of the area of interest that is covered by at least *k*-sensors. If *k* = 3 and some location is covered by only 2 sensors, that particular location does not count towards the 3-coverage;
- **Tracking Time Mean Percentage** (also referred to as **Mean Tracking Time**) is the mean percentage of the total time in which the network can track the target (*i.e.*, the target can be sensed by at least three sensors and those sensors can send messages towards the sink);
- **Mean Tracking Error** is the average tracking error (*i.e.*, the Euclidean distance between the target's real and estimated positions); and
- **Residual** is the measure for the localization confidence, as discussed in Section 3.1.

### 5.2. Simulation Results

#### 5.2.1. Impact of Localization and Density Control Algorithms on 3-Coverage

**Figure 9.**
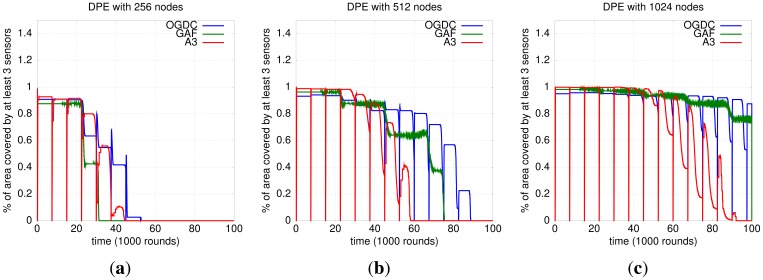
Localization, Density Control and 3-Coverage. (**a**) DPE; *n* = 256; (**b**) DPE; *n* = 512; (**c**) DPE; *n* = 1024.

Both OGDC and A3 run in rounds that we define as density control rounds (not to be confused with simulation rounds). In the context of density control algorithms, a round is the time interval between two executions of the algorithm. In the end of each round, all nodes return to a state where a new topology can be created. As a consequence, we observe a periodic drop on 3-coverage for OGDC and A3. That does not happen with GAF because each node sends periodic messages to the sensors in the cell to determine if there is a node responsible for that cell. The result is that GAF shows smaller discontinuities, as shown in Figure 9.

Our results show that OGDC is the algorithm that performs best at maintaining 3-coverage, no matter what localization mechanism is used. Figure 9 shows its superiority when DPE is used; the same behavior is observed with RPE.

**Figure 10.**
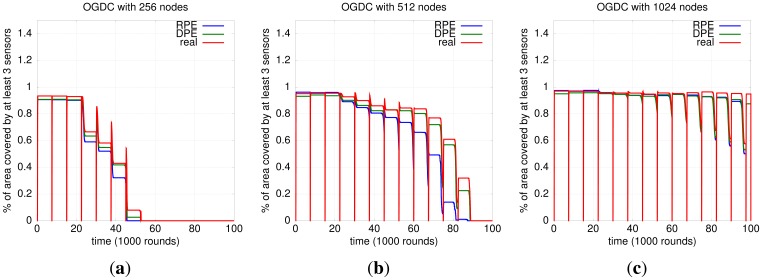
Localization and 3-Coverage of OGDC. (**a**) OGDC; *n* = 256; (**b**) OGDC; *n* = 512; (**c**) OGDC; *n* = 1024.

To determine which localization algorithm leads to a better 3-coverage with OGDC, we compare their behaviors. Figure 10 shows that OGDC gives better 3-coverage when used with the actual localization. Since perfect (actual) localization is infeasible and serves only as a baseline, we conclude that the combination DPE-OGDC is the most suited for maintaining the 3-coverage of a network, among the algorithms evaluated.

#### 5.2.2. Impact of Localization and Density Control Algorithms on Target Tracking

##### Localization

**Figure 11.**
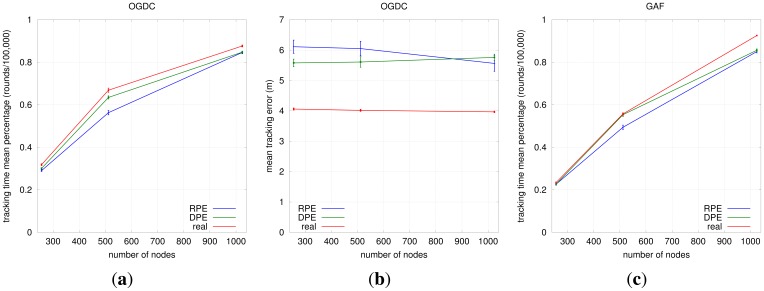

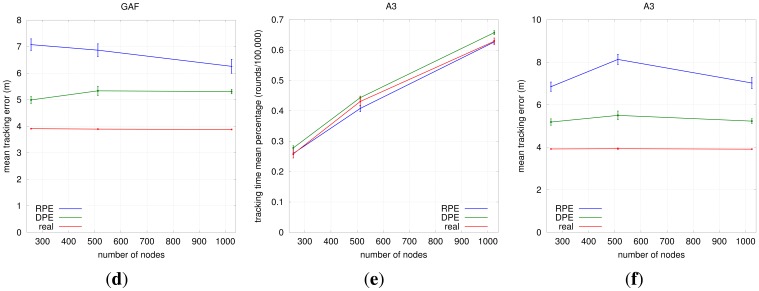
Choosing the Localization Algorithm. (**a**) Mean Tracking Time (OGDC); (**b**) Mean Tracking Error (OGDC); (**c**) Mean Tracking Time (GAF); (**d**) Mean Tracking Error (GAF); (**e**) Mean Tracking Time (A3); (**f**) Mean Tracking Error (A3).

Our results show that the choice of the localization algorithm impacts on the quality of target tracking, which is independent of the density control algorithm, as shown in Figure 11. DPE results in smaller average tracking error than RPE (actual localization is once again used as a baseline). The difference between the two algorithms in terms of tracking time is not statistically relevant; we work with 99% confidence intervals.

##### Density Control

**Figure 12.**
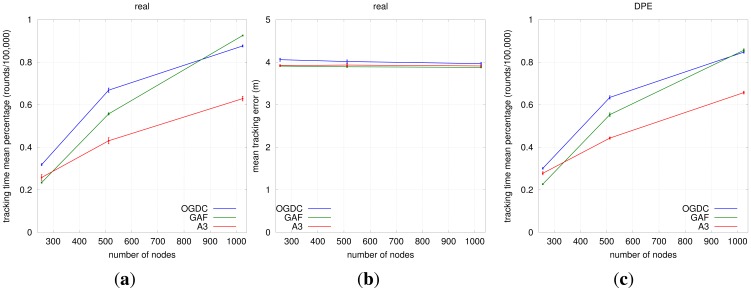

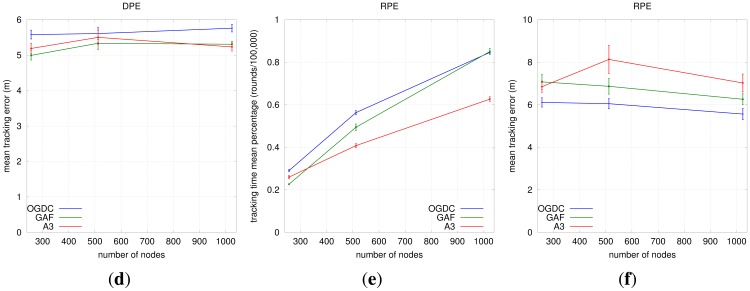
Choosing the Density Control Algorithm. (**a**) Mean Tracking Time (Real); (**b**) Mean Tracking Error (Real); (**c**) Mean Tracking Time (DPE); (**d**) Mean Tracking Error (DPE); (**e**) Mean Tracking Time (RPE); (**f**) Mean Tracking Error (RPE).

The choice for the density control algorithm depends in fact on the network density. In low densities (e.g., 0.05 sensors/m^2^), OGDC and A3 have similar performance; there is an overlap of the confidence intervals of both mean tracking error and mean tracking time, as shown in Figure 12(c) and 12(d). When we increase the node density to 0.1 sensors/m^2^, OGDC is clearly superior in terms of tracking time while the three algorithms show equivalent results when it comes to tracking error. Finally, as the density gets even higher (e.g., 0.2 sensors/m^2^), compared to OGDC, GAF gives smaller tracking errors and similar tracking time.

Figure 12 is actually misleading; it seems that GAF can track a target for longer than OGDC in high density networks. However, in such high density scenarios, all density control algorithms maintain 3-coverage above 60% for more than 100,000 rounds, which is our simulation time. Since GAF does not turn off all nodes periodically like OGDC and A3, it seems to outperform OGDC's tracking time when *n* = 1024. However, the GAF network will eventually run out of battery faster than OGDC, and the latter will be able to track the target for a longer time, just like we observe with *n* = 256 and *n* = 512.

Our results show that A3 is not a scalable solution for target tracking; it only performs well (in comparison to OGDC and GAF) in low density networks. Therefore, we do not recommend its use for target tracking applications. When used with DPE, GAF results in slightly smaller mean tracking error than OGDC and is equivalent to A3. However, it is outperformed by OGDC in terms of mean tracking time. Given that the difference on tracking error between GAF and OGDC is small (about 0.5m) and OGDC keeps the network alive for longer than GAF, we conclude that OGDC is the best choice for target tracking applications among the three algorithms.

#### 5.2.3. Impact of the Residual on Density Control and Target Tracking

The results in Table 1 indicate that incorporating the residual into GAF has a negative impact on tracking performance (mean tracking time and mean tracking error). Therefore, we do not recommend such practice with GAF.

Our version of A3 that considers the residual while creating the network topology performs better than the original version. With RPE, our modification yields greater mean tracking time and smaller mean tracking error. However, that advantage applies only to the tracking time when DPE is the localization algorithm; the modified algorithm produces statistically equivalent results in comparison to the residual-unaware version.

Adapting OGDC to use the residual does not result in significant difference; we observe overlap of the confidence intervals for both tracking error and tracking time, with both localization algorithms. Consequently, we cannot confirm nor deny the hypothesis that incorporating the residual into OGDC results in better tracking performance.

**Figure 13.**
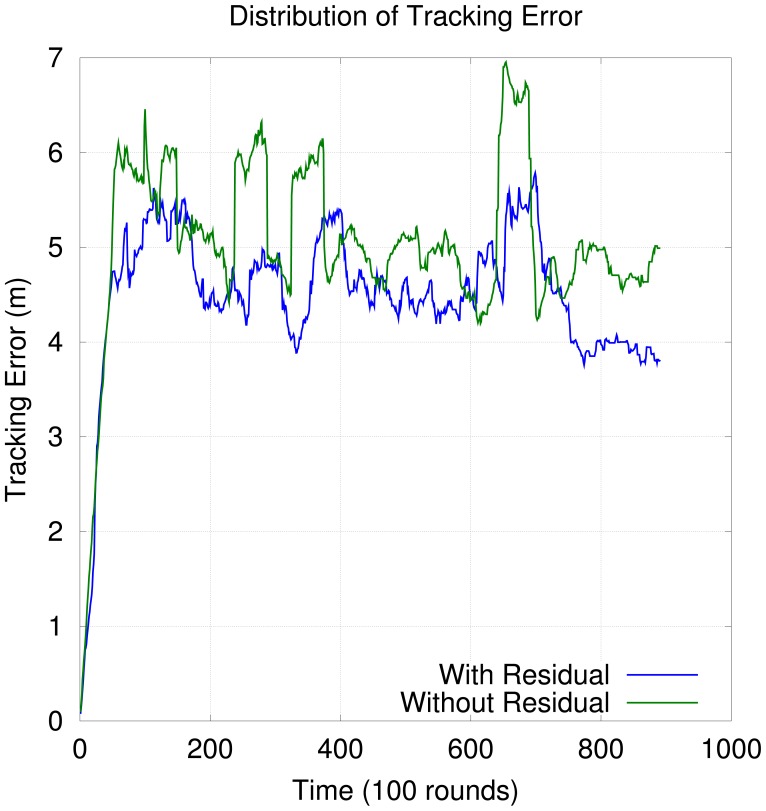
Distribution of Tracking Error Over Time with OGDC.

Figure 13 shows the distribution of tracking error over time when the residual is considered in OGDC. In the beginning of the simulation, the tracking error is smaller because the first few topologies are based on the nodes with smallest residuals. However, as time goes by, the network keeps reorganizing itself until nodes with higher residual start to participate in the network. As a consequence, tracking error increases. Although Figure 13 gives the impression that it is a good idea to incorporate the residual into OGDC, the difference between OGDC with residual and OGDC without residual in terms of tracking performance is not statistically significant, as shown in Table 1.

#### 5.2.4. Impact of *k*-Coverage

**Figure 14.**
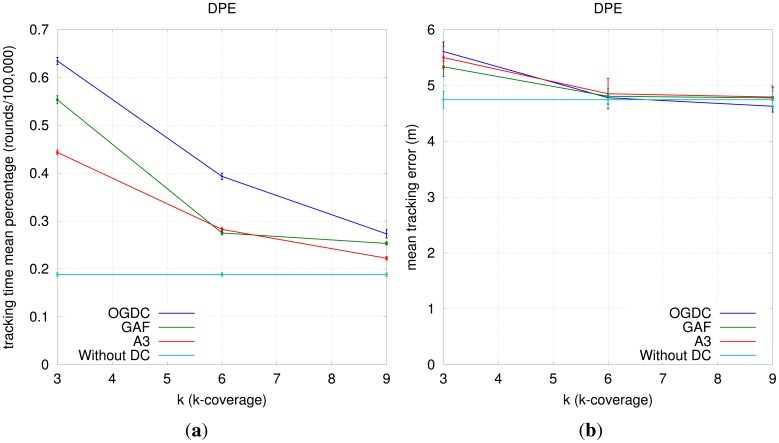
Impact of *k* on Target Tracking. (**a**) Mean Tracking Time (DPE); (**b**) Mean Tracking Error (DPE).

Our results show that increasing the number of sensors capable of sensing the target does not increase tracking precision. In addition, increasing *k* forces the density control algorithms to turn off less nodes, resulting in shorter tracking time. Figure 14 shows the effect of increasing *k* when DPE is used; we observe same behavior for actual localization and RPE. Therefore, we conclude that increasing *k* is not recommended.

Figure 14 also shows how the network behaves without density control; we do not observe higher tracking precision and the tracking time becomes even smaller than when *k* = 9. The graph shows a lower bound for tracking time as we increase *k*.

## 6. Final Remarks

This work provides an empirical foundation to determine which combination of localization and density control algorithms should be used in a target tracking application. We have made extensive simulation of several combinations of such algorithms and we firmly believe our contribution is an important one.

We started by evaluating the combined impact of localization algorithms (DPE and RPE) and density control algorithms (OGDC, GAF, and A3) on the maintenance of 3-coverage of a network. In such scenario, the network can be used for target tracking applications. We also evaluated how the combinations of those algorithms affect both tracking error and tracking time. Finally, we investigated if tuning the density control algorithms with parameters like *k*-coverage and residual usage has a positive impact on target tracking applications.

Previous results show that DPE is better than RPE for target tracking applications [3]. Our results show that this conclusion also applies to scenarios where a density control algorithm is used to increase the network lifetime. The choice of such algorithm has little impact on the tracking error, but significantly influences the tracking time.

A3 performed well in sparse networks and poorly in dense networks. Since it has a serious scalability issue, A3 is not recommend for target tracking applications. GAF's advantage is that it does not turn off the whole network from time to time, as opposed to OGDC and A3. As a result, while most sensors still have energy and when the network density is high, GAF manages to track the target more often than OGDC. However, as time goes by and the sensors start to run out of battery, OGDC is better at keeping the 3-coverage and consequently manages to track the target for longer time. Therefore, we conclude that among the density control algorithms we evaluated, OGDC is the most suited for target tracking applications.

We also investigated whether modifying the density control algorithms to be residual-aware increases the tracking performance. The impact is negative on GAF, positive on A3 and neutral on OGDC. Since we do not recommend A3 for target tracking applications and incorporating the residual into OGDC and GAF yields no gain in performance, we do not recommend such practice for density control algorithms in target tracking applications.

Finally, we observed how increasing *k*-coverage impacts on target tracking. Our results do not show considerable gain in tracking accuracy. However, increasing *k* significantly decreases the mean tracking time. As a result, we do not recommend using *k* > 3 for target tracking.

Current work leads to some interesting future directions. Our results are based exclusively on simulations; it is necessary to validate them with actual sensors. All the scenarios we evaluated had a single target and fixed sensors. It is important to evaluate the tracking performance with mobile sensors and multiple targets. Finally, our analysis considers that 3-coverage is necessary for target tracking. However, there are less precise tracking methods that do not require so. It is interesting to extend our analysis to such methods. We are also working on defining the theoretical performance bounds when such algorithm combinations are integrated.

## Figures and Tables

**Figure 5.**
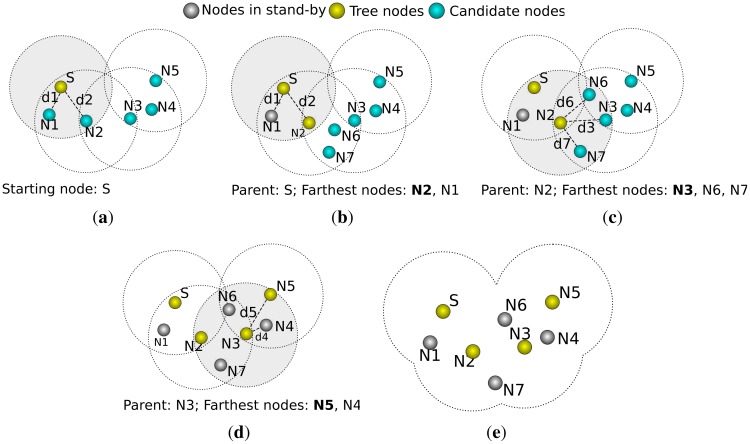
Overview of *A3*. (**a**) Nodes 1 and 2 are candidates; (**b**) Node 1 sleeps, 2 is chosen; (**c**) Node 2 acts as a parent; (**d**) Nodes 3 and 6 sleep, node 5 is chosen; (**e**) CDS tree nodes are defined.

**Table 1.** Use of Residual in Density Control.

**Combination**	**Res.**	**Mean Error (m)**	**Mean Time (%)**
DPE-OGDC	no	5.6083 ± 0.2305	0.6347 ± 0.0098
DPE-OGDC	yes	5.5105 ± 0.2157	0.6491 ± 0.0137
RPE-OGDC	no	6.0508 ± 0.3209	0.5630 ± 0.0136
RPE-OGDC	yes	5.6463 ± 0.2207	0.5770 ± 0.0252
DPE-GAF	no	5.3350 ± 0.2343	0.5535 ± 0.0111
DPE-GAF	yes	5.2835 ± 0.2290	0.3538 ± 0.0149
RPE-GAF	no	6.8673 ± 0.4966	0.4943 ± 0.0204
RPE-GAF	yes	8.0791 ± 0.4672	0.2332 ± 0.0130
DPE-A3	no	5.5008 ± 0.2736	0.4436 ± 0.0067
DPE-A3	yes	5.4096 ± 0.2507	0.4837 ± 0.0144
RPE-A3	no	8.1320 ± 0.8846	0.4078 ± 0.0131
RPE-A3	yes	6.5698 ± 0.4677	0.4580 ± 0.0033
